# Strategies for Enhancing the Stability of Lithium Metal Anodes in Solid-State Electrolytes

**DOI:** 10.3390/mi15040453

**Published:** 2024-03-28

**Authors:** Hanbyeol Lee, Taeho Yoon, Oh B. Chae

**Affiliations:** 1 School of Chemical, Biological and Battery Engineering, Gachon University, Seongnam-si 13120, Republic of Korea; byulbbang99@gachon.ac.kr; 2 Department of Chemical Engineering, Kyung Hee University, Yongin-si 17104, Republic of Korea

**Keywords:** Li-ion batteries, Li metal, energy density, solid-state electrolytes

## Abstract

The current commercially used anode material, graphite, has a theoretical capacity of only 372 mAh/g, leading to a relatively low energy density. Lithium (Li) metal is a promising candidate as an anode for enhancing energy density; however, challenges related to safety and performance arise due to Li’s dendritic growth, which needs to be addressed. Owing to these critical issues in Li metal batteries, all-solid-state lithium-ion batteries (ASSLIBs) have attracted considerable interest due to their superior energy density and enhanced safety features. Among the key components of ASSLIBs, solid-state electrolytes (SSEs) play a vital role in determining their overall performance. Various types of SSEs, including sulfides, oxides, and polymers, have been extensively investigated for Li metal anodes. Sulfide SSEs have demonstrated high ion conductivity; however, dendrite formation and a limited electrochemical window hinder the commercialization of ASSLIBs due to safety concerns. Conversely, oxide SSEs exhibit a wide electrochemical window, but compatibility issues with Li metal lead to interfacial resistance problems. Polymer SSEs have the advantage of flexibility; however their limited ion conductivity poses challenges for commercialization. This review aims to provide an overview of the distinctive characteristics and inherent challenges associated with each SSE type for Li metal anodes while also proposing potential pathways for future enhancements based on prior research findings.

## 1. Introduction

Since the commercialization of lithium (Li)-ion batteries (LIBs) in the 1990s, they have been widely applied in various fields, such as small electronic devices, Electric Vehicles (EVs), and Energy Storage Systems (ESSs), owing to their high energy density and stable lifespan [1,2,3,4]. Graphite, which is mainly used as the anode material in current LIBs, demonstrates the reversible intercalation/de-intercalation of Li ions between graphene layers, providing stable charge and discharge cycles. However, the theoretical capacity of graphite is limited to approximately 372 mAh/g, posing challenges for high-capacity energy storage [5,6,7,8]. Transition metal oxides (e.g., MnO_2_, ZnO, Fe_2_O_3_, and SnO_2_) also have gained widespread use as electrode materials in the field of LIBs due to their more substantial theoretical capacities than graphite. Nevertheless, poor cycling performance owing to low electric conductivity and structural collapse as result of large volume expansion during lithiation/delithiation results in poor rate performance and a limited lifespan for LIBs [9]. To overcome this limitation, researchers worldwide are exploring next-generation anode materials for energy storage, including silicon and Li metal.

As Li metal is the lightest member of the alkali metal group and has the lowest reduction voltage (−3.04 V vs. standard hydrogen electrode), extensive research has been reported on highly promising next-generation anode materials [10,11,12]. However, the use of Li metal in liquid electrolytes faces many challenges owing to its high reactivity to nonuniform Li dendrite formation, which results in hazardous safety concerns (e.g., fire and explosion). Most of the graphite surface is covered by the reductive decomposition products of electrolyte components created during the initial few cycles. In the case of graphite, owing to the small volume change during the charge and discharge cycle, solid electrolyte interface (SEI) crack generation is mitigated, which minimizes the exposure of the fresh graphite surface during cycling, preventing continuous electrolyte decomposition. On the contrary, the initial SEI of Li metal is generated as soon as the Li metal is exposed to the electrolyte since the electrochemical potential of Li metal is lower than the potential window for the electrolyte components [13,14,15]. The uncontrolled Li deposition causes the inhomogeneous exposure of fresh Li metal, resulting in low cycling performance and Li dendrite formation.

To overcome the critical issues associated with Li metal, various attempts have been made to suppress Li dendrite growth by using electrolyte additives [16], forming a protective layer [17], and changing the Li salt [18]. However, these approaches present notable challenges in the practical implementation of Li metal batteries. Incorporating solid-state electrolytes (SSEs) into all-solid-state lithium-ion batteries (ASSLIBs) has promise as a method to replace traditional liquid electrolytes. This transition in physical composition suppresses dendrite growth, with SSEs serving dual roles as separators [19,20]. Therefore, when Li metal meets SSEs, the convergence of Li metal with SSEs offers the potential for high energy density and exceptional safety in ASSLIBs.

To realize highly promising ASSLIBs, SSEs should satisfy the following requirements: (1) high ion conductivity at room temperature, (2) a wide electrochemical window, and (3) high chemical compatibility with the Li metal and cathode [21]. They are typically classified into two main categories: inorganic and polymeric forms. Inorganic SSEs can be further categorized into sulfide and oxide types. Understanding the electrochemical characteristics and properties of these three materials is crucial owing to their differences. This review focuses on examining the characteristics and challenges associated with sulfide, oxide, and polymer SSEs when paired with lithium metal, in addition to potential solutions based on previous research.

## 2. Sulfide SSEs

Sulfide SSEs have demonstrated superior ionic conductivities compared with other solid electrolytes. The high ion conductivity of sulfide SSEs, which is similar to that of organic liquid electrolytes, makes them promising electrolytes for solid-state batteries (Li_10_GeP_2_S_12_, 1.2 × 10^−^^3^ S cm^−^^1^ [22]; Li_2_S-P_2_S_5_, 1.7 × 10^−^^2^ S cm^−^^1^ [23]). In recent years, Kanno et al. doped the Li_10_GeP_2_S_12_-type structure LSiPSBrO and obtained Li_9.54_[Si_0.6_Ge_0.4_]_1.74_P_1.44_S_11.1_Br_0.3_O_0.6_, the bulk ion conductivity at room temperature of which was determined to be 3.2 × 10^−^^2^ S cm^−^^1^ [24]. Han et al. demonstrated a different type of sulfide SSE (Li_7_Si_2_S_7_I) created using an ordering of sulfide and iodide [25]. It built a fast transport path of diverse lithium coordination geometries and anion neighbors (1.01 × 10^−^^2^ S cm^−^^1^). However, the inherent limitations of sulfide SSEs, such as insufficient dendrite suppression, pose challenges, leading to potential short circuits [26]. Furthermore, their narrow electrochemical window can lead to various reactions with Li metal [27,28], emphasizing the importance of understanding dendrite growth mechanisms and interfacial reactions.

### 2.1. Challenges of Sulfide SSEs for Li Metal

**Figure 1:**
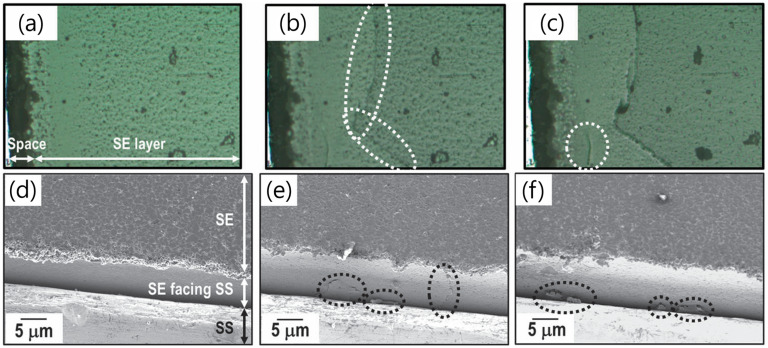
Optical microscope images of the solid electrolyte (SE) layer and the interface between SE and stainless steel (SS): (**a**) before the test; (**b**) Li deposition at the interface at 2 mA cm^−2^ for 730 s and (**c**) at 100 mA cm^−2^ for 2 h. SEM images of the interface between the SE layer and SS at different positions on the same cell (**d**) before and (**e**) after Li deposition for 600 s and (**f**) 1920 s [29]. White arrows showed cracks formed during the charge and discharge process, while black arrows showed inhomogeneous plating of Li. Reprinted with permission from Ref. [29]. Copyright 2013 Royal Society of Chemistry.

In liquid electrolytes, Li-ion deposition occurs uniformly owing to consistent contact with Li metal, resulting in an even current density and ion deposition. In contrast, sulfide SSEs exhibit a localized high current density and Li-ion deposition owing to point contact with the Li metal electrode. Consequently, Li dendrite growth and crack formation along crystal boundaries have been reported (Figure 1) [29]. Porz et al. revealed that Li was preferentially deposited on existing cracks or defects, inducing crack-tip stresses and propagation beyond the critical current density [30]. Zhang et al. demonstrated that alloy materials such as lithium–indium (Li-In) alloys are widely used at the laboratory scale because of their (electro)chemical stability; however, Li-In dendrites grow along the pores and grain boundaries, with high compactness of the Li_6_PS_5_Cl SSE layer during the charge/discharge process [31].

**Figure 2:**
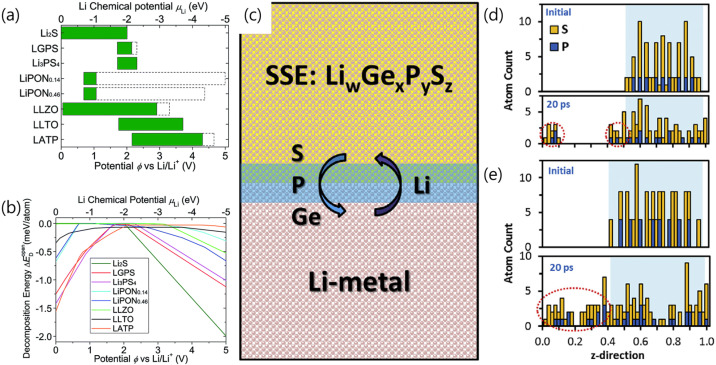
(**a**) Electrochemical window and (**b**) decomposition energy [27]. Reprinted with permission from Ref. [27]. Copyright 2013 Royal Society of Chemistry. (**c**) Schematic representation of interfacial phenomena between sulfide SSEs and Li metal anode. Sulfur and phosphorus atom profiles along z-direction for (**d**) Li_7_P_3_S_11_-(100)/Li-slab and (**e**) Li_2_P_2_S_6_-(100)/Li slab [32]. At 20 ps, some peaks moved outside the SSE space, which means that some S and P are present at SSE/Li metal interface. Reprinted/adapted with permission from Ref. [32]. Copyright 2018 ELSEVIER.

In addition, the strong reducing power of Li metal can trigger various reactions at the Li metal–sulfide SSE interface (Figure 2a,b) [27]. In Figure 2a,b, for example, the electrochemical windows of Li_10_GeP_2_S_12_ and Li_3_PS_4_ are narrow compared to other SSEs. Thus, Li_10_GeP_2_S_12_ and Li_3_PS_4_ begin to be lithiated and reduced at 1.71 V. Computational studies by Forero et al. using the density functional theory (DFT) and ab initio molecular dynamics (AIMD) demonstrated that sulfide SSE anions (such as PS_4_^3−^, P_2_S_6_^2−^, P_2_S_7_^4−^, and GeS_4_^4−^) react with Li to form Li-S, Li-P, and Li-Ge species (Figure 2c–e) [32]. These studies have demonstrated that sulfide SSEs generate byproducts such as Li_3_P, Li_2_S, and Li_15_Ge_4_ during discharge to 0 V, which causes the decomposition of SSEs. Wenzel et al. used XPS to identify compounds formed in sulfide-based SSEs, with Li_7_P_3_S_11_ exhibiting the lowest resistance among the studied SSEs, resembling the values found in liquid electrolytes [33]. Argyrodite-type sulfide-based SSEs produce SEI layers comprising Li_3_P, Li_2_S, and decomposed LiX (X = Cl or Br) [34].

### 2.2. Strategies to Overcome Disadvantages of Sulfide SSEs for Li Metal

**Figure 3:**
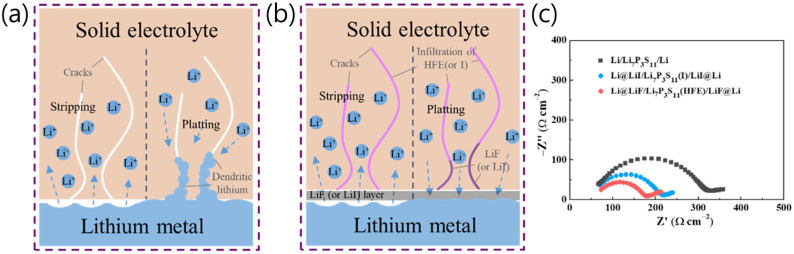
Schematic diagrams of the Li stripping and plating behavior of (**a**) bare Li metal with an untreated SSE and (**b**) LiF- or LiI-coated Li metal with HFE- or I-infiltrated electrolyte. (**c**) Nyquist plots of symmetric cells [21]. Reprinted with permission from Ref. [21]. Copyright 2018 ELSEVIER.

Wang et al. used a LiF (or LiI) layer at the interface between Li metal and a sulfide SSE and penetrated the sulfide SSE with methoxyperfluorobutane (HFE) (or an I solution) to suppress Li dendrite growth [21]. Li dendrite formation in sulfide SSEs is closely related to the interface stability of the electrolyte with Li metal. The LiF and LiI layers were electrochemically stable in both solid electrolytes and Li metal. The LiF (or LiI) interface layer ensured stability/compatibility at the Li metal/sulfide SSE interface and simultaneously inhibited Li’s dendritic growth (Figure 3a,b). Consequently, the interfacial resistance of the cells using LiF- and LiI-coated Li metals was effectively reduced (Figure 3c).

**Figure 4:**
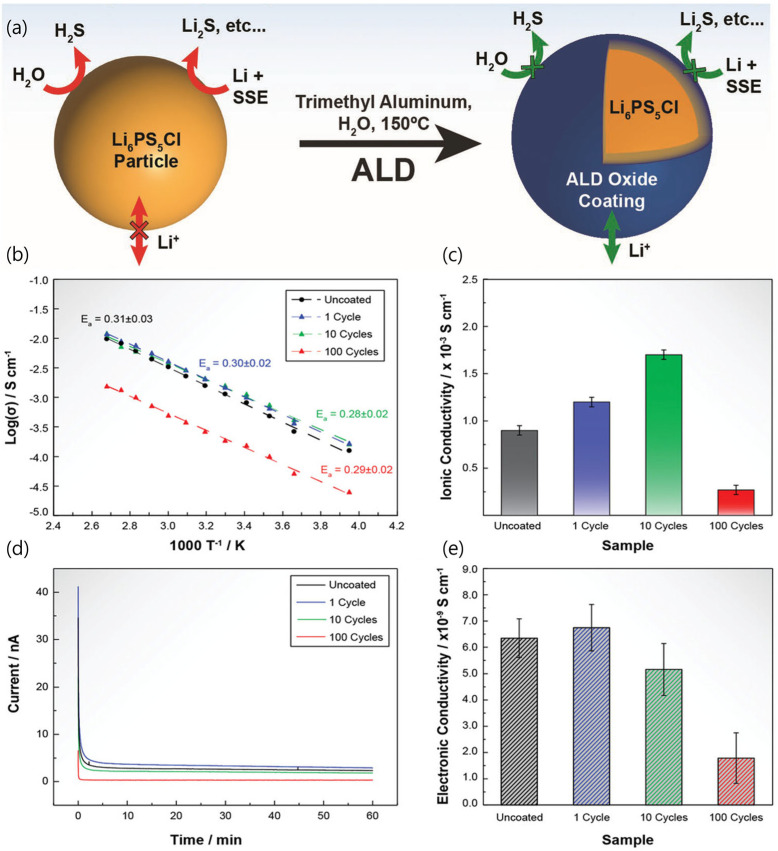
(**a**) Schematic of the coating strategy based on ALD to produce oxide−coated Li_6_PS_5_Cl powders. (**b**) Arrhenius plots, (**c**) ionic conductivity at 25 °C, (**d**) current versus time (DC polarization at 200 mV; 25 °C), and (**e**) electronic conductivity at 25 °C for Li_6_PS_5_Cl pellets pressed from powders coated by 1, 10, and 100 ALD alumina cycles in comparison to pellets pressed from uncoated powders [35]. Reprinted with permission from Ref. [35]. Copyright 2023 Wiley-VCH.

To mitigate dendrite formation and other undesirable reactions, it is crucial to minimize sub-reactions between Li metal and SSEs and SEI formation. The introduction of an artificial SEI has shown promise for inhibiting dendrite growth and ensuring uniform Li deposition. Connell et al. utilized Atomic Layer Deposition (ALD) to create a coating layer on Li_6_PS_5_Cl sulfide-based SSE powders, resulting in improved stability and a two-fold increase in ion conductivity (Figure 4) [35]. In addition, ALD is a method of depositing thin films which relies on sequential gas-phase chemical processes. It falls within the broader category of Chemical Vapor Deposition (CVD). The enhanced ion conductivity was attributed to a decrease in the Arrhenius activation energy owing to the coating. Additionally, optimizing the coating thickness is essential to prevent hindered Li^+^ conductivity caused by excessively thick Al_2_O_3_ layers.

Furthermore, altering the composition of sulfide SSEs is an effective strategy. Composition tuning has proven to be effective in enhancing stability and controlling decomposition products, thereby preventing material propagation and reducing interfacial resistance. Sulfide SSE tuning is categorized into cation substitution and anion substitution, depending on the atoms comprising the structure. In a previous study, research was conducted to improve the performance of sulfide SSEs through cation substitution. Substituting a cation (e.g., Ge with Si, Sn, Al, P, Ba, Zn, or Y in LGPS) can adjust the stability of sulfide SSEs with minimal impact on material properties such as ion conductivity, as indicated by first-principles calculations [36]. However, this study demonstrates that Si, Sn, and Ge are also unstable versus Li and tend to form non-passivating degradation products on the Li metal. Research has also been conducted on anion substitution in addition to cation substitution. The partial substitution of an anion, especially sulfur by oxygen, has been shown to increase the thermodynamic stability of Li_10_GeP_2_S_12_ by providing stronger chemical bonding, and oxygen substitution was recently used to stabilize the Li_10_GeP_2_S_12_-phase of Li_3_PS_4_ [37,38].

## 3. Oxide SSEs

Oxide SSEs exhibit substantial energy gaps between their valence and conduction bands which provide enhanced stability at elevated voltages. In addition, the ionic mobility of oxide SSEs surpasses that of polymer electrolytes. Among the various oxide SSEs, garnet-type SSEs have the highest ion conductivity. The garnet-type Li_7_La_3_Zr_2_O_12_ (LLZO) oxide SSE synthesized by Weppner et al. is particularly attractive because of its good stability against Li metal, large electrochemical window, and easy handling in ambient environments [39,40,41]. The LLZO SSEs exhibit the best resistance to Li reduction and have the lowest reduction potential (approximately 0.05 V vs. Li/Li^+^) and the most stable interface.

### 3.1. Challenges of Oxide SSEs for Li Metal

**Figure 5:**
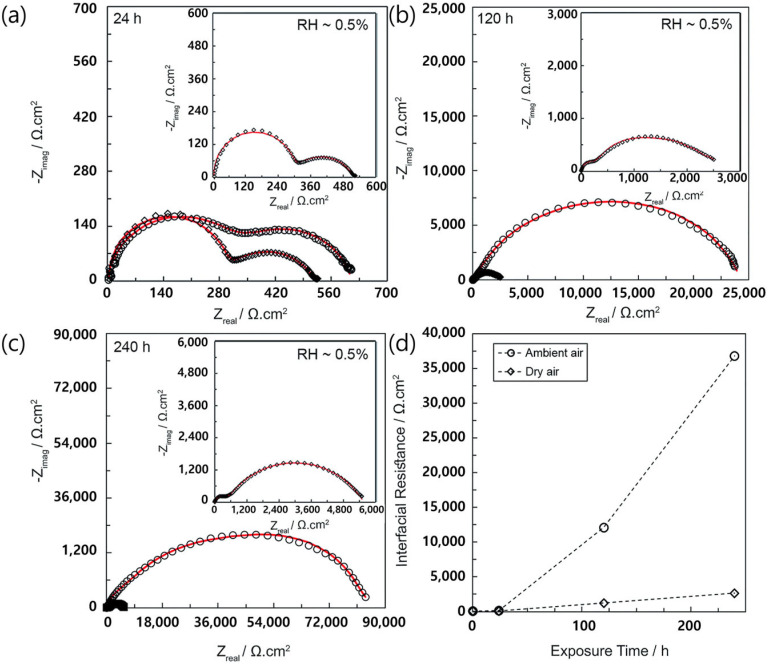
Impedance spectra measured at room temperature for Li metal symmetric cells after various exposure times to ambient (○) and dry air (◇). Schematic depicting the asymmetric Li metal symmetric cell before and after air exposure and equivalent circuit model used for fitting EIS data at (**a**) 24 h, (**b**) 120 h, and (**c**) 240 h. The insets display the impedance spectra for Li-LLZO-Li for samples exposed to dry air. (**d**) The LLZO/Li metal interfacial resistance versus time after exposure to ambient and dry air [43]. Reprinted with permission from Ref. [43]. Copyright 2017 Royal Society of Chemistry.

Several studies have shown that the absorbed CO_2_ reacts directly with Li in the garnet structure to form Li_2_CO_3_ (Equation (1)) [42]. The Li_2_CO_3_ on the surface of the Li metal increases the interfacial resistance and decreases the ion conductivity (Figure 5) [43]. Cheng et al. reported that the large impedance at the LLZO/Li metal interface is not inherent to the material; however, it originates from the Li_2_CO_3_ due to ambient air exposure [44].

(1) $${Li}_{7}{La}_{3}{Zr}_{2}O_{12}+x\cdot {CO}_{2}\to {Li}_{7-2x}{La}_{3}{Zr}_{2}O_{12}+x\cdot {Li}_{2}{CO}_{3}$$

**Figure 6:**
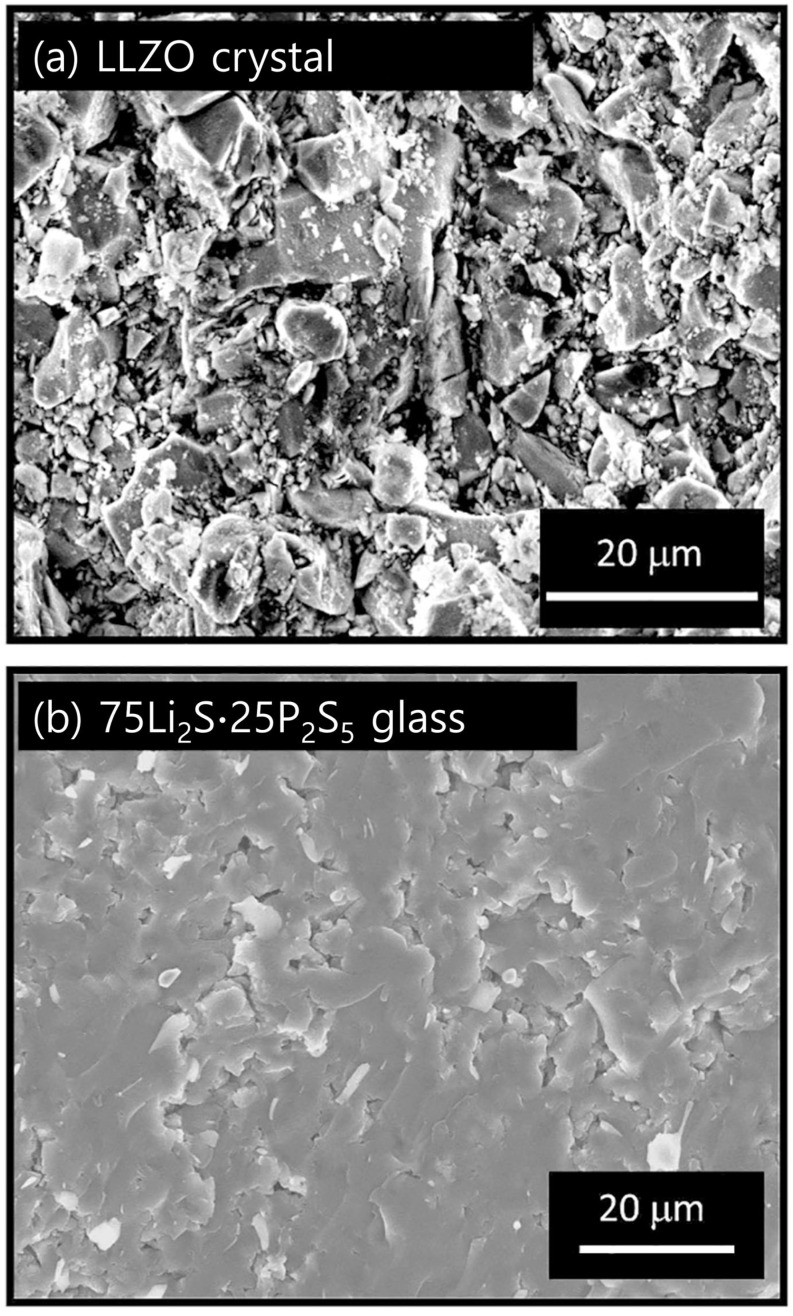
Fracture cross-section SEM images of (**a**) LLZO and (**b**) sulfide electrolyte particles glass-pelletized at room temperature [45]. Reprinted with permission from Ref. [45]. Copyright 2013 Nature Publishing Group.

Another interface problem is the low wettability between garnet-type oxide SSEs and Li metal. When LLZO SSEs were manufactured through compression at room temperature, the particles did not agglomerate, and void spaces were present within the resulting structures (Figure 6) [45]. The rigid connection between the electrode and the SSEs and the existence of an inert surface layer of Li_2_CO_3_ resulted in poor interface compatibility, which led to an interfacial impedance of 10^2^~10^3^ Ω cm^2^ and thus unstable Li plating and stripping [46,47].

### 3.2. Strategies to Overcome Disadvantages of Oxide SSEs for Li Metal

**Figure 7:**
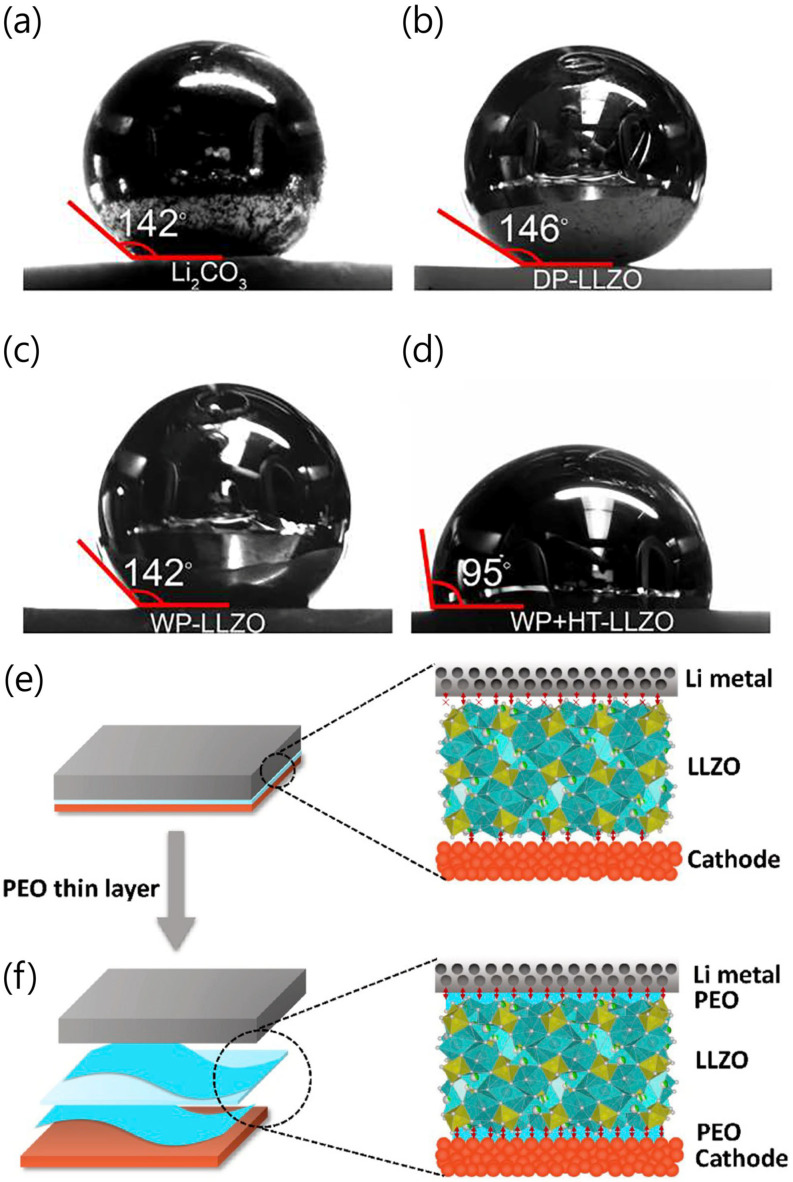
Contact angle measurements of molten metallic on (**a**) Li_2_CO_3_, (**b**) dry polishing of LLZO, (**c**) wet polishing of LLZO, and (**d**) wet polishing of LLZO after heat treatment at 500 °C [51]. Reprinted with permission from Ref. [51]. Copyright 2017 American Chemical Society. Schematic of electrode–LLZO interface for solid-state battery. (**e**) Hard-to-hard interfaces with bad contact between electrodes and LLZO. (**f**) Soft-to-hard interfaces with improved connection [52]. Reprinted with permission from Ref. [52]. Copyright 2020 ELSEVIER.

Many studies have reported methods to eliminate Li_2_CO_3_, a factor that increases interfacial resistance (e.g., by carbothermal reactions, high-temperature calcination, or acid treatment) [48,49,50]. Furthermore, both dry and wet polishing have been reported to be effective approaches for removing Li_2_CO_3_ from a surface [13,51]. Sakamoto et al. noted that among the two approaches, dry and wet polishing, the latter was mentioned as the more effective method for removing Li_2_CO_3_ (Figure 7a–d) [51].

A common strategy for overcoming point contacts with Li metal is to use an artificial interface layer. The excellent flexibility and softness of polymers can enhance the low interface contact between LLZO and Li metal. Flexible polymers can serve as coatings for LLZO and electrodes, resembling the characteristics found in liquid electrolytes, and are advantageous for scalable processing. Yang et al. developed a thin polyethylene oxide (PEO) buffer layer to modify LLZO (Figure 7e,f) [52]. In their study, the interfacial resistance decreased from 1360 Ω cm^2^ to 175 Ω cm^2^ and exhibited good stability to Li metal at 0.2 mA cm^−^^2^. PEO-Poly(acrylamide-2-methyl-1-propane-sulfonate) (PAS)-coated Li_6.5_La_3_Zr_1.5_Ta_0.5_O_12_ (LLZTO) was demonstrated by Zhou et al. [53]. PAS is a Li^+^-conducting polymer which carries out the transportation of high amounts of Li^+^. The interfacial impedance of Li/LLZTO/Li was around 5000 Ω, while a Li/PEO-PAS/LLZTO/PEO-PAS/Li cell exhibited an interfacial impedance of less than 400 Ω. Owing to good Li ion transport and contact with Li metal, the capacity retention was around 137 mAh/g at 0.2 C after 160 cycles.

**Figure 8:**
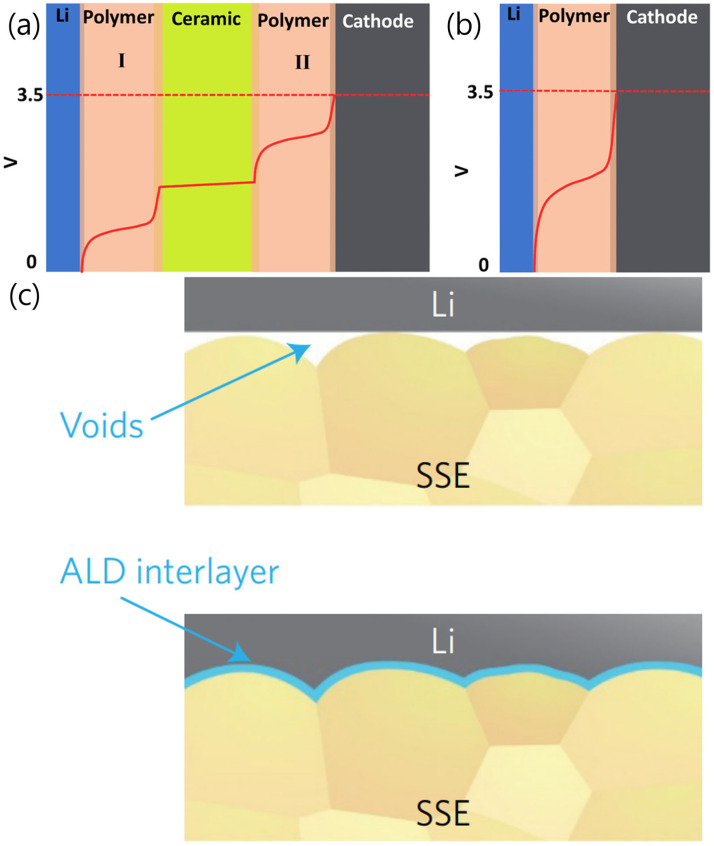
Schematic of the electric potential profile across the (**a**) sandwich electrolyte and (**b**) individual polymer electrolyte in the charge process of a full cell [54]. Both polymer I and II represent CPMEA. Reprinted with permission from Ref. [54]. Copyright 2016 American Chemical Society. (**c**) Schematic of the behavior of LLZO with molten Li metal [55]. Reprinted with permission from Ref. [55]. Copyright 2017 Nature Publishing Group.

In addition, Goodenough et al. designed a cell utilizing a cross-linked polymer, poly(ethylene glycol) methyl ether acrylate (CPMEA), as a buffer layer between the cathode and Li metal (Figure 8a,b) [54]. The polymer layer not only improved contact at the interface because of its flexibility and softness but also increased the Li^+^ flux, thereby improving the cycling performance and preventing dendrite formation in the cell.

Similar to the approach described for sulfide SSEs, there is a method employing ALD to form a thin film at the Li metal and LLZO interface, effectively improving wettability and reducing resistance. Hu et al. demonstrated that the wettability of SSEs against molten Li was significantly improved by applying an alumina ALD coating [55]. The LLZO/Li metal interfacial resistance decreased from 1710 Ω cm^2^ to 34 Ω cm^2^ owing to Li metal on a lithiated pure alumina layer with a varied Li stoichiometry, Li_x_Al_2_O_3+x/2_; a strong chemical bond with the Li metal formed, and it exhibited good ion conductivity (Figure 8c). Various metals and metal oxides, including Si, ZnO, and Au, have been reported in studies in which ALD was employed for coating. These materials demonstrated enhanced wettability with molten Li and concurrently reduced interfacial resistance [56,57,58].

## 4. Polymer SSEs

Polymer SSEs are predominantly composed of thermally stable polymers, such as the previously mentioned polymers PEO, poly(vinylidene fluoride) (PVDF), and polyacrylonitrile (PAN). Among these, PEO, which can form various salts through interactions between ether oxygen atoms and cations, has been extensively studied as a dry polymer electrolyte host. Its ability to dissolve various salts makes it the most widely studied dry polymer electrolyte host. Consequently, they can be utilized in the design of all-solid-state batteries with self-supporting films without altering current battery assembly processes [59]. In addition, gel-formulated polymer electrolytes have been reported as various types of polymer solid-state electrolytes. In this configuration, the liquid components within the gel facilitate ion conduction. In this review, no detailed exposition is provided on this subject.

### 4.1. Challenges of Polymer SSEs for Li Metal

**Figure 9:**
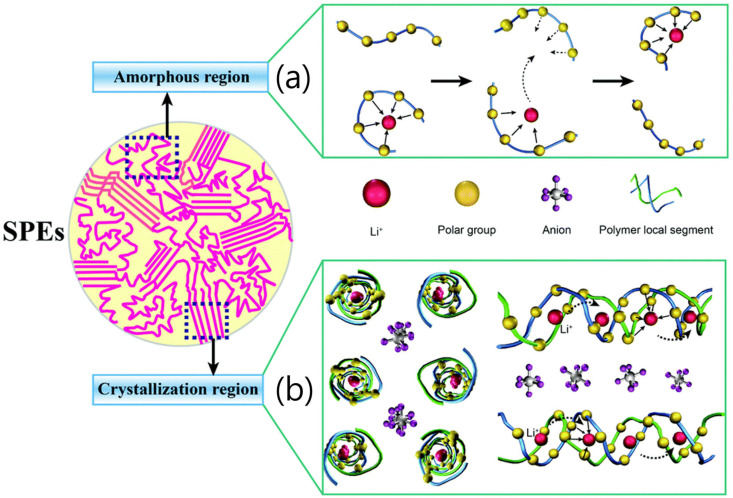
Schematic of conduction mechanism in two kinds of polymer electrolytes. (**a**) Amorphous polymer electrolytes and (**b**) crystalline polymer electrolytes [62]. Reprinted with permission from Ref. [62]. Copyright 2016 Royal Society of Chemistry.

PEO-based electrolytes generally exhibit low ion conductivity at an order of 10^−^^8^~10^−^^7^ S cm^−^^1^ at room temperature because the ion conduction in the PEO-based electrolyte mainly occurs in the amorphous part of the PEO matrix, while the crystalline part provides very limited ion motion (note that the PEO chain is mainly crystalline below 65 °C) [60,61]. The Li^+^ ion conduction mechanisms of both the amorphous and crystalline polymers are shown in Figure 9 [62].

**Figure 10:**
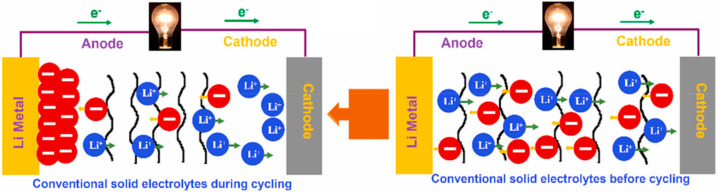
The operational phenomena of conventional SPEs [64]. Reprinted with permission from Ref. [64]. Copyright 2021 ELSEVIER.

Moreover, most polymer electrolytes are dual-ion conductors in which Li^+^ and its counter anions are both mobile. Therefore, Li^+^ is coupled with Lewis basic sites in the polymer matrix; thus, Li^+^ is usually less mobile than anions. Therefore, the Li^+^ transference number of ion conductors with dual ions is generally lower than 0.5. Li^+^; in addition, counter anions move in opposite directions during discharge cycles, leading to the accumulation of anions at the anode side and causing concentration gradients and cell polarization (Figure 10) [63,64]. The transference number is defined as the ratio of the electric current derived from the cation to the total electric current. If the number is close to one, it implies that the ion-conducting performance of the polymer electrolyte is mainly accomplished by the cation [65].

### 4.2. Strategies to Overcome Disadvantages of Polymer SSEs for Li Metal

**Figure 11:**
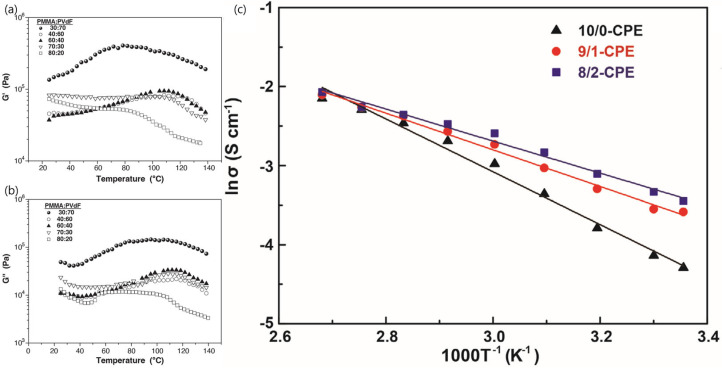
Temperature sweep test from 25 to 140 °C of (**a**) G′ vs. temperature and (**b**) G″ vs. temperature [67]. Reprinted with permission from Ref. [67]. Copyright 2006 ELSEVIER. (**c**) The ion conductivity of DOL and TOM copolymer electrolytes in a temperature range of 25–110 °C (black, red, and blue lines represent the DOL/TOM molar ratio as 10/0, 9/1, and 8/2, respectively) [70]. Reprinted with permission from Ref. [70]. Copyright 2020 Wiley-VCH.

Among the various methods available, polymer blending is the most feasible. Polymer blending involves mixing at least two polymers, with or without chemical bonding [66]. Poly(methyl methacrylate) (PMMA)- and PVDF-blended polymer electrolytes were demonstrated by Nicotera et al. [67]. Oscillatory rheological tests showed better mechanical properties for the intermediate composition of the blend (Figure 11a,b). Subsequently, Ganessan et al. [49] studied the design of blended polymer electrolytes using machine learning [68]. Electrolyte performance was measured using a combination of ionic transport and electrolyte mechanical properties. In this study, through the utilization of a machine learning approach known as Bayesian optimization, a trade-off between ion transport and electrolyte mechanical properties was identified as a function of various design parameters, including host molecular weight and polarity.

Copolymer electrolytes may be considered a method for reducing crystalline PEO. A copolymer is a polymer composed of multiple monomer species. Copolymer electrolytes exhibit conductivities exceeding 10^−^^4^ S cm^−^^1^ at room temperature which result from the highly amorphous structure of the studied polymer hosts and their high flexibility, as evidenced by a T_g_ value below 210 K [69]. Recently, Li et al. reported the synthesis of copolymers through cationic ring-opening polymerization using 1,3-dioxolane (DOL) and trioxymethylene (TOM) as monomers [70]. The ion conductivity from the study by Li et al. reached 3.56 × 10^−4^ S cm^−1^ at room temperature (Figure 11c).

**Figure 12:**
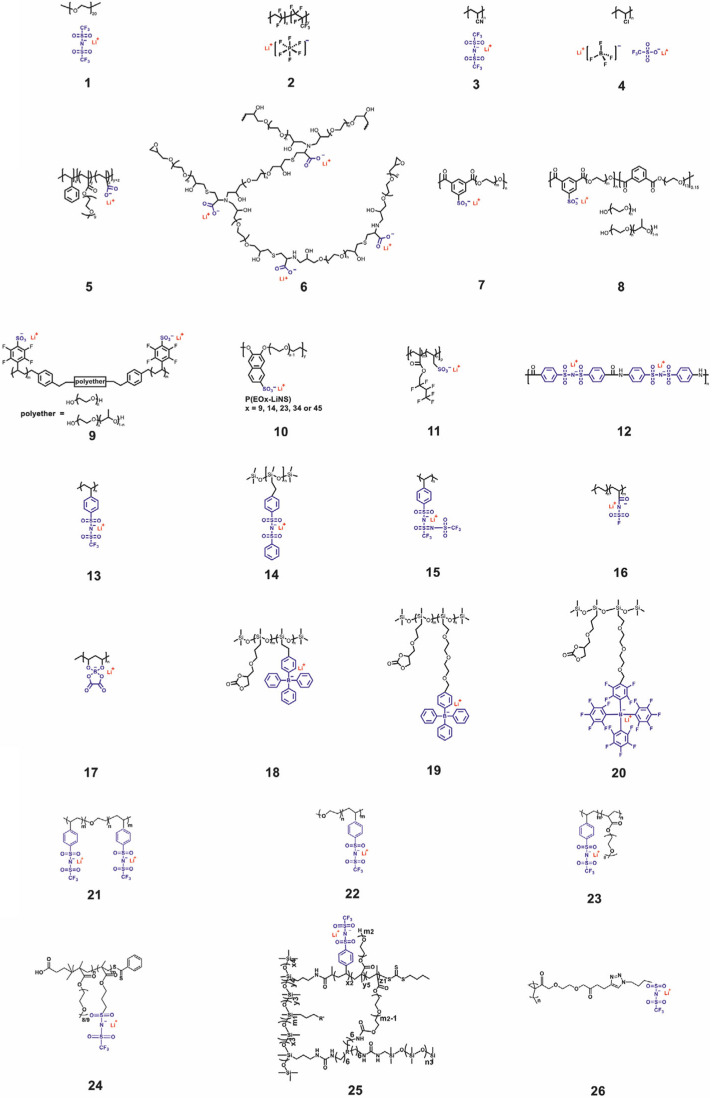
Representative structures of the reported SICPEs [77]. Reprinted with permission from Ref. [77]. Copyright 2021 Wiley-VCH.

The use of single−ion conducting polymer electrolytes (SICPEs), ideally defined as polymer electrolytes with Li^+^ transference numbers similar to unity (approximately 1), can be an effective method for reducing concentration polarization. With a higher transference number than traditional polymer electrolytes, SICPEs exhibit remarkably decreased polarization, which should theoretically allow them to suppress Li dendrites [71,72,73,74]. Conventional SICPEs are fabricated by covalently bonding anions to the polymer backbone, allowing only single-ion species to be mobile. Gohy et al. reported a copolymer electrolyte combining a single-ion conducting anionic group (poly(lithium methacrylate-*co*-oligoethylene glycol methacrylate)) [75]. Its ion conductivity reached up to 2 × 10^−5^ S cm^−1^. Mecerreyes et al. developed SICPEs based on UV-cross-linkable polyurethanes [76]. A coulombic efficiency near 100% and capacity retention exceeding 72.8% were measured after 80 cycles at 0.1 C. The ion conductivities and properties of the other SICPEs are displayed in Figure 12 and Table 1 [77].

## 5. Conclusions

In this review, the properties, challenges, and previous studies of sulfide, oxide, and polymer SSEs for Li metal anodes were examined. Despite the high theoretical capacity and energy density of lithium metal, its commercialization is challenging when using liquid electrolytes. This challenge has led to extensive research on SSEs. Due to the varying physical and chemical properties of each SSE, the challenges and improvement strategies for Li metal anodes also differ. At the interfaces between sulfide SSEs and lithium metal, challenges such as lithium dendrite formation and side reactions are prevalent. Various methods can be employed to tackle these issues, including applying interlayers, utilizing ALD coatings, and optimizing the electrolyte composition. In the case of oxide SSEs, significant hurdles arise from substantial interfacial resistance due to point contact with lithium metal and impurity generation from reactions with CO_2_. These challenges can be alleviated through techniques such as polishing, employing buffer layers, and implementing ALD coatings similar to those used for sulfide SSEs. Polymer SSEs encounter challenges associated with low ionic conductivity and concentration polarization, which can potentially lead to lithium dendrite formation. Addressing these issues involves strategies like polymer blending, copolymerization, and the utilization of solid ion-conducting polymer electrolytes. Despite these efforts, numerous technical and commercial challenges remain in the commercialization of solid-state electrolytes (SSEs). However, with a thorough understanding of SSEs and ongoing research on Li metal anodes and cathodes, the commercialization of ASSLIBs is expected to be achieved.

## Figures and Tables

**Table 1:** Summary of various SICPEs with corresponding property parameters [77]. Reused with permission from Ref. [77]. Copyright 2021 Wiley-VCH.

Materials	T_g_ [°C]	t_Li_^+^	Ionic Conductivity [S cm^−1^]	Testing Temperature [°C]	Mechanical Strength [MPa]
1	−57	0.29	N/A	N/A	N/A
2	N/A	0.46	8.9 × 10^−4^	25	N/A
3	−105.2	N/A	2.5 × 10^−3^	23	N/A
4	N/A	N/A	2.6 × 10^−3^	25	N/A
5	N/A	Close to unity	≈10^−8^	20	N/A
6	<0	0.86	1.2 × 10^−4^	85	N/A
7	−37	N/A	10^−6^	25	N/A
8	−20	N/A	≈10^−5^	25	N/A
9	<0	N/A	1.4 × 10^−5^	60	N/A
10	−38	N/A	5.5 × 10^−6^	120	N/A
11	17	0.92	10^−4^	80	7.1
12	N/A	0.92	1.4 × 10^−4^	25	N/A
13	152	N/A	≈10^−5^	41	N/A
14	−110	0.89	7.2 × 10^−4^	25	5.8
15	−47	>0.9	7.6 × 10^−6^	25	N/A
16	−30	0.91	5.84 × 10^−4^	25	N/A
17	N/A	N/A	6.11 × 10^−6^	25	N/A
18	10	N/A	10^−8.2^	25	N/A
19	−17	N/A	10^−7.0^	25	N/A
20	−16	N/A	10^−6.9^	25	N/A
21	N/A	0.85	1.35 × 10^−5^	60	10
22	N/A	0.95	3.8 × 10^−4^	90	N/A
23	<0	>0.9	10^−4^	60	N/A
24	−61	0.83	2.3 × 10^−6^	25	N/A
25	−17.5	0.79	4.5 × 10^−7^	30	0.37
26	−27 to −14	0.79–0.99	10^−5–^10^−4^	90	N/A
